# Heart failure with preserved ejection fraction: Calculating the risk of future heart failure events and death

**DOI:** 10.3389/fcvm.2022.921132

**Published:** 2022-10-21

**Authors:** Lore Schrutka, Benjamin Seirer, René Rettl, Theresa-Marie Dachs, Christina Binder, Franz Duca, Daniel Dalos, Roza Badr-Eslam, Johannes Kastner, Christian Hengstenberg, Florian Frommlet, Diana Bonderman

**Affiliations:** ^1^Department of Internal Medicine II, Division of Cardiology, Medical University of Vienna, Vienna, Austria; ^2^Center for Medical Statistics, Informatics and Intelligent Systems, Institute of Medical Statistics, Medical University of Vienna, Vienna, Austria; ^3^Department of Internal Medicine V, Division of Cardiology, Clinic Favoriten, Vienna, Austria

**Keywords:** heart failure, heart failure with preserved ejection fraction, risk stratification, prediction model, outcome, survival

## Abstract

**Objective:**

We sought to develop a clinical model to identify heart failure patients with preserved ejection fraction (HFpEF) at highest risk for acute HF events or death.

**Methods and results:**

Between 2010 and 2019, 422 patients with HFpEF were followed. Acute HF events occurred in 190 patients (45%), including 110 (58%) with recurrent hospitalizations. Those with recurrent events had worse 6-min walk test (*p* < 0.001), higher brain N-terminal prohormone natriuretic peptide (NT-proBNP, *p* < 0.001), and higher New York Heart Association functional class (NYHA, *p* < 0.001). Overall survival rates in patients with 1 HF event vs > 1 HF events were: at 1-year 91.6 vs. 91.8%, at 3-years 84.7 vs. 68.3% and at 5-years 67.4 vs. 42.7%, respectively (*p* < 0.04). The Hfpef survivAL hOspitalization (HALO) score revealed best predictive capability for all-cause mortality combining the variables age (*p* = 0.08), BMI (*p* = 0.124), NYHA class (*p* = 0.004), need for diuretic therapy (*p* = 0.06), left atrial volume index (*p* = 0.048), systolic pulmonary artery pressure (*p* = 0.013), NT-proBNP (*p* = 0.076), and number of prior hospitalizations (*p* = 0.006). HALO score predicted future HF hospitalizations in an ordinal logistic regression model (OR 3.24, 95% CI: 2.45–4.37, *p* < 0.001). The score performance was externally validated in 75 HFpEF patients, confirming a strong survival prediction (HR 2.13, 95% CI: 1.30–3.47, *p* = 0.002).

**Conclusions:**

We developed a model to identify HFpEF patients at increased risk of death and HF hospitalization. NYHA class and recurrent HF hospitalizations were the strongest drivers of outcome.

## Introduction

Heart failure incidence with preserved ejection fraction (HFpEF) is steadily increasing and is associated with a significant risk of fatal and non-fatal cardiovascular events, including hospitalization for acute HF (1). Until recently there were no evidence-based medical treatments proven to affect disease progression or prevent hospitalizations, leaving HFpEF a major clinical challenge (2–4). Management of HFpEF is particularly complicated by disease heterogeneity and the confounding of diagnosis by comorbid medical conditions (5). Impaired ventricular relaxation along with increased diastolic stiffness represents the central disturbance in HFpEF leading to increased intracardiac filling pressures, exercise intolerance and ultimately fluid retention (6). As a result, the cardiovascular system shows a heightened sensitivity to the smallest hemodynamic changes and may decompensate with increasing load, leading to a rapid onset of pulmonary edema requiring hospitalization. Therefore, current treatment strategies aim to improve clinical status by controlling blood pressure and body fluid (7).

Despite intensive efforts, episodes of acute HF remain one of the main reasons for hospitalization in people over 65 years (8). Hospitalizations are critical events in the course of chronic HF, with hospitalized patients subsequently having a higher mortality rate (9).

Recently, the use of sacubitril/valsartan has been shown to modestly, though not statistically significant, reduce hospitalization rates for HF in HFpEF and is now the first U.S. Food and Drug Administration-approved drug for the treatment of HFpEF (4). Moreover, treatment with the sodium-glucose co-transporter 2 (SGLT2) inhibitor empagliflozin has now demonstrated to improve outcomes in HFpEF patients, primarily through a reduction in HF hospitalizations comparable to that previously reported in patients with HF and reduced ejection fraction (10). In light of current advances in treatment options, it is even more critical to identify patients at increased risk for recurrent HF events and mortality. Therefore, the aim of this study was to derive a clinical model to identify HFpEF patients at high risk for acute HF events or death.

## Materials and methods

### Subjects and study design

Consecutive patients presenting with HFpEF between December 2010 and December 2019 were included and prospectively followed within an observational registry established at the Department of Cardiology of the Medical University of Vienna. Written informed consent was collected from all patients before enrolment in the institutional registry. The study protocol complied with the Declaration of Helsinki and was approved by the local Ethics Committee (EK #796/2010).

### Clinical definitions

HFpEF was diagnosed according to the current consensus statement of the European Society of Cardiology (11) and the American College of Cardiology Foundation/American Heart Association task force (12). The following diagnostic criteria had to be fulfilled for study inclusion: (1) clinical signs and symptoms of HF, (II) echocardiographic left ventricular (LV) ejection fraction ≥ 50%, (3) evidence of diastolic LV dysfunction, and (4) serum N-terminal pro–B-type natriuretic peptide (NT-proBNP) concentrations ≥220 pg/ml (11). The diagnosis of HFpEF was confirmed by right heart catheterization when pulmonary artery wedge pressure exceeded 15 mmHg (13). Baseline evaluation included physical examination, 12-lead electrocardiogram, laboratory assessment including serum NT-proBNP measurement, transthoracic echocardiography, six-minute walk tests and coronary angiography combined to right heart catheterization. NT-proBNP measurements at baseline were performed in heparin plasma using the Elecsys system (Roche Diagnostics, Mannheim, Germany) according to the standard procedures of the local laboratory. In patients with an episode of acute HF, NT-proBNP levels were determined on admission using the accredited standards of the local laboratory of the respective hospital and at the time of first follow-up after discharge.

### Outcome measures and follow-up

Patients were prospectively followed by outpatient visits and/or telephone calls at 6-month intervals. The primary outcome measure of this analysis was overall survival within the follow-up period. The secondary outcome measure was hospitalization due to acute worsening of HF, defined by the concomitant presence of the symptom of sudden-onset dyspnea and clinical signs of acute cardiac decompensation, including weight gain and fluid retention, with the presence of pulmonary or peripheral edema requiring intravenous diuresis.

### Statistical analysis

Continuous data were described via median and interquartile range (IQR), whereas discrete data were summarized using absolute and relative frequencies. Mann–Whitney U-test or Kruskal Wallis 1-way ANOVA were used to compare the distribution of continuous variables between different groups. Associations between categorical variables were assessed using the Chi-square test or Fisher’s exact test. The longitudinal measurements of NT-proBNP levels at the time of administration and after discharge of different HF hospitalizations were analyzed with linear mixed models using the R package lme4 (14). Due to strong right skewness, NT-proBNP levels were first log-transformed before fitting mixed models. A random intercept was used for each patient to model within-patient correlation. In addition, NT-proBNP values at the time of admission were included as covariates in the linear mixed model.

Based on medical considerations, the following 16 clinical variables were preselected as potential predictors of all-cause mortality: sex, age, body mass index (BMI), functional class of the New York Heart Association (NYHA), 6-minute walk distance (6MWD), comorbidities such as atrial fibrillation, type two diabetes mellitus, chronic kidney disease, chronic obstructive pulmonary disease (COPD), anemia, use of loop diuretics, echocardiographic parameters such as the left atrial volume index (LAVI), right atrial volume, systolic pulmonary arterial pressure (sPAP), serum levels of NT-proBNP and gamma-glutamyltransferase. Additional variables of interest related to hospitalizations before baseline were the number of hospitalizations before baseline (categorized as no, one, or more than one hospitalization) and the time interval between the last hospitalization and baseline. The predictive model with the acronym HALO (Hfpef survivAL hOspitalization) score was obtained via augmented backward elimination for Cox proportional hazards methods (15, 16) using the R package abe version 3.0.1 (17). The HALO score was then used to fit an ordinal logistic regression model to predict the number of HF hospitalizations after baseline. External validation of the risk calculator was performed using data from 75 independent patients diagnosed with HFpEF, fulfilling the same inclusion and exclusion criteria as the derivation cohort but did not undergo hemodynamic evaluation, enrolled at our institution between April 2011 and June 2021 and followed the recommendations of Royston and Altman (18). Overall survival of the validation cohort was stratified by 1st and 3rd quartiles of the HALO score, with Kaplan–Meier plots providing visual comparison of discrimination.

In general, computations were performed with SPSS version 26.0 (IBM SPSS, Armonk, NY, USA) and R version 3.6.0 (R Foundation for Statistical Computing, Vienna, Austria). We considered a two-sided significance level alpha = 0.05 for statistical testing.

## Results

### Baseline characteristics and heart failure hospitalizations

Of the 422 HFpEF patients included in this analysis, 190 patients (45%) experienced HF hospitalizations during a median follow-up time of 42 (IQR 23–72) months. A graphical representation of the study is depicted in Supplementary Figure 1. Of those hospitalized, 110 (58%) had recurrent hospitalizations with a median frequency of 3 admissions (IQR: 2–4). There were 8 patients with more than 4 hospitalizations before baseline (maximum: 8) and 18 patients with more than 4 hospitalizations after baseline (maximum: 14). Comparison between stable patients, patients with one hospitalization, and patients with recurrent events revealed significant differences in performance on 6MWT (*p* < 0.001), levels of NT-proBNP (*p* < 0.001), and the proportion of NYHA functional class ≥ III (*p* < 0.001), as well as the need for diuretic therapy at baseline (*p* < 0.001; Table 1). Compared to those with only one event, patients with recurrent hospitalizations had substantially worse performance on the 6MWT (*p* = 0.027), higher levels of NT-proBNP (*p* < 0.001), and a higher proportion of NYHA functional class ≥ III (*p* = 0.013). Further differences are summarized in Table 1 and co-medication of patients in Supplementary Table 1.

**TABLE 1:** Baseline characteristics of registered patients with one or recurrent episodes of acute heart failure requiring hospitalization.

	Stable patients (*n* = 232)	Patients with one HF hospitalization^†^ (*n* = 80)	Patients with recurrent HF hospitalizations^†^ (*n* = 110)	*P*-value^‡^	*P*-value 1 vs > 1 HF hospitalizations^$^
**Clinical parameters**					
Age, years (IQR)	73 (72–74)	75 (71–76)	73 (72–75)	0.613	0.637
Female gender, *n* (%)	168 (72.4)	59 (73.8)	70 (63.6)	0.192	0.140
Body mass index (kg/m^2^) (IQR)	28 (25–30)	29 (29–31)	30 (29–32)	**0.032**	0.216
6-minute walk distance (m) (IQR)	380 (360–406)	320 (306–365)	289 (250–311)	**<0.001**	**0.027**
NYHA functional class ≥ III, *n* (%)	113 (49.8)	51 (66.2)	88 (82.2)	**<0.001**	**0.013**
NT-proBNP (pg/ml) (IQR)	770 (615–860)	1046 (935–1210)	1959 (1579–2184)	**<0.001**	**<0.001**
HF hospitalization prior to study inclusion, *n* (%)	–	45 (56.3)	87 (79.1)	–	**0.001**
Loop diuretic therapy, *n* (%)	90 (39.0)	57 (71.3)	90 (81.8)	**<0.001**	0.086
**Co-morbidities**					
Arterial hypertension, *n* (%)	213 (92.2)	77 (96.3)	106 (96.4)	0.207	0.967
Atrial fibrillation, *n* (%)	121 (52.4)	54 (67.5)	73 (66.4)	**0.011**	0.870
Diabetes mellitus, *n* (%)	60 (26.0)	30 (37.5)	54 (49.1)	**<0.001**	0.112
Chronic kidney disease*, *n* (%)	92 (42.4)	48 (61.5)	76 (71.0)	**<0.001**	0.175
Anemia, *n* (%)	79 (34.1)	32 (40.0)	73 (66.4)	**<0.001**	**<0.001**
Sleep apnea, *n* (%)	13 (5.6)	3 (3.8)	12 (10.9)	0.096	0.071
Chronic obstructive pulmonary disease, *n* (%)	49 (21.2)	22 (27.5)	44 (40.0)	**0.001**	0.074
**Laboratory parameters**					
Hemoglobin (g/dL) (IQR)	12.8 (11.8–13.5)	12.6 (11.2–14.0)	11.7 (10.8–12.8)	**<0.001**	**0.005**
Serum iron (μg/dL) (IQR)	77 (56–99)	68 (52–105)	54 (40–75)	**<0.001**	**0.002**
Albumin (g/L) (IQR)	41.6 (39.3–43.7)	41.1 (38.7–43.8)	40.3 (37.1–42.8)	**0.003**	0.081
ASAT (U/L) (IQR)	24 (20–30)	25 (21–32)	23 (20–32)	0.816	0.518
ALAT (U/L) (IQR)	21 (16–28)	22 (17–28)	19 (14–27)	0.084	0.084
Gamma-GT (U/L) (IQR)	29 (19–47)	38 (22–90)	57 (28–88)	**<0.001**	0.104
GFR (ml/min/1.73 m^2^) (IQR)	64.25 (42.76–77.83)	55.51 (40.18–67.70)	49.83 (38.36–63.77)	**<0.001**	0.058
Blood urea nitrogen (mg/dL) (IQR)	18.95 (14.45–24.35)	25.40 (20.10–32.75)	25.00 (18.90–36.10)	**<0.001**	0.883
HbA1c (%) (IQR)	5.8 (5.5–6.2)	5.9 (5.5–6.4)	6.1 (5.6–6.9)	**0.003**	0.175
**Echocardiographic parameters**					
Left atrial diameter (mm) (IQR)	60 (60–62)	61 (60–64)	64 (63–66)	**<0.001**	**0.035**
Left atrial volume index (ml/m^2^) (IQR)	38 (35–39)	39 (33–44)	40 (37–54)	**0.047**	0.149
Left ventricular end diastolic diameter (mm) (IQR)	44 (40–47)	43 (39–46)	44 (40–48)	0.312	0.225
LV-ejection fraction (%) (IQR)	60 (54–64)	59 (54–65)	60 (55–66)	0.675	0.523
E/E’ ratio (IQR)	12.8 (10.4–15.3)	13.4 (10.3–17.8)	15.9 (9.7–18.8)	0.202	0.588
E/A ratio (IQR)	1.17 (0.81–1.63)	1.13 (0.90–2.30)	1.90 (1.03–2.88)	**0.002**	0.100
Right atrial diameter (mm) (IQR)	59 (53–65)	60 (56–68)	64 (58–70)	**<0.001**	**0.018**
RA volume (ml) (IQR)	57 (53–61)	65 (51–74)	70 (62–80)	**<0.001**	**0.016**
Right ventricular end diastolic diameter (mm) (IQR)	35 (31–40)	35 (31–40)	40 (33–44)	**<0.001**	**0.002**
TAPSE (mm) (IQR)	19 (15–22)	18 (15–22)	17 (13–20)	**0.017**	0.180
Moderate and severe tricuspid insufficiency, *n* (%)	113 (50.2)	46 (62.2)	78 (71.6)	**0.001**	0.182
Pulmonary arterial systolic pressure (mmHg) (IQR)	48 (37–59)	56 (44–69)	66 (51–79)	**<0.001**	**0.001**
**Invasive hemodynamic parameters**					
Mean pulmonary arterial pressure, mmHg (IQR)	30 (24–35)	32 (25–39)	38 (31–44)	**<0.001**	**0.001**
Right atrial pressure (mmHg) (IQR)	11 (7–13)	11 (7–16)	14 (10–18)	**<0.001**	**0.006**
Pulmonary artery wedge pressure (mmHg) (IQR)	18 (18–20)	19 (18–21)	22 (20–23)	**<0.001**	**0.008**
Left ventricular end diastolic pressure (mmHg) (IQR)	18 (17–19)	18 (17–20)	21 (20–23)	**0.002**	**0.005**

NYHA, New York Heart Association; NT-proBNP, N-terminal prohormone of brain natriuretic peptide; HF, heart failure; ALAT, alanin aminotransferase; ASAT, aspartat aminotransferase; Gamma-GT, gamma-Glutamyl Transferase; GFR, glomerular filtration rate; LDH, lactatdehydrogenase; HbA1c, glycated hemoglobin; LA, left atrial; RA, right atrial; LV, left ventricular; E/E’, ratio of peak early transmitral flow velocity to peak early diastolic mitral annulus velocity; E/A, ratio of peak early transmitral flow velocity to mitral peak velocity of late filling; TAPSE, tricuspid annular plane systolic excursion. Values are given as median and interquartile range (IQR), or total numbers (n) and percent (%). Bold indicates *p* < 0.05.

*Estimated glomerular filtration rate <60 ml/min/1.73 m^2^.

^†^HF hospitalization was defined by the concomitant presence of the symptom of sudden-onset dyspnea and clinical signs of acute cardiac decompensation, including weight gain and fluid retention, with presence of pulmonary or peripheral edema requiring intravenous diuresis.

^‡^For comparisons of stable, patients with one HF hospitalization and patients with recurrent HF hospitalizations Chi-square test was used for categorical and Kruskal wallis 1-way ANOVA for continuous variables.

^$^For comparisons of patients with one HF hospitalization and patients with recurrent HF hospitalizations Chi-square test was used for categorical and Mann–Whitney U-test for continuous variables.

### Clinical course of patients with recurrent hospitalizations

**FIGURE 1:**
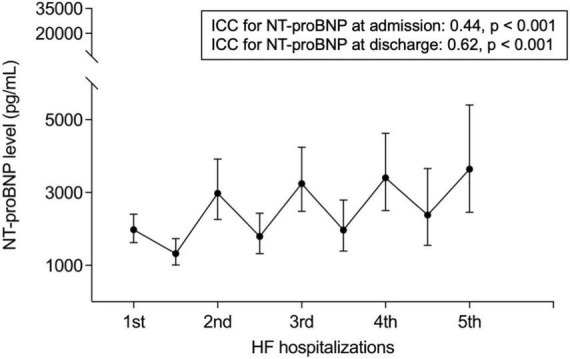
NT-proBNP levels during the course of recurrent HF hospitalizations. ICC stands for intraclass correlation coefficient.

Evaluation of NT-proBNP levels at the time of hospitalization and during follow-up showed a strong deflection of the biomarker at the time of presentation with acute HF, which, however, did not return to baseline upon reaching a stable state and continued to rise with each hospitalization (Figure 1). Fitting linear mixed models for the log-transformed longitudinal data with a random intercept for each patient confirmed a significant positive trend with each consecutive hospitalization both for NT-proBNP levels at the time of admission (Slope = 0.13, *p* < 0.001) and after discharge (Slope = 0.16, *p* < 0.001). NT-proBNP levels after discharge were dependent on levels at the time of admission for each hospitalization event. Adding the NT-proBNP level at the time of admission as a covariate offsets this dependence and supports the assumptions of the mixed model. The increase in NT-proBNP after discharge remains significant (Slope = 0.08, *p* < 0.001) when correcting for NT-proBNP levels at the time of admission. This would suggest that with each consecutive HF hospitalization, NTproBNP is less likely to return to pre-admission levels.

**FIGURE 2:**
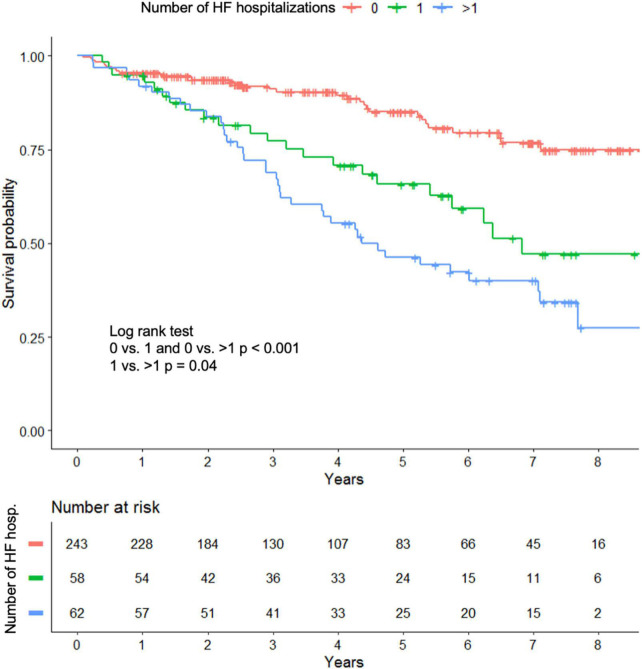
Kaplan–Meier curves for all cause death in patients with heart failure hospitalization and stable patients.

Over the observation period, 107 patients (25%) died. There was a strong association between survival and the number of hospitalizations after baseline, with Kaplan–Meier curves showing shorter survival in patients with recurrent HF events (1-year survival: 1 HF event 91.6% vs. > 1 HF events 91.8%, 3-years survival: 1 HF event 84.7% vs. > 1 HF events 68.3%, 5-years survival: 1 HF event 67.4% vs. > 1 HF events 42.7%, *p* = 0.04; Figure 2).

### Risk calculator for survival and future heart failure hospitalizations

**FIGURE 3:**
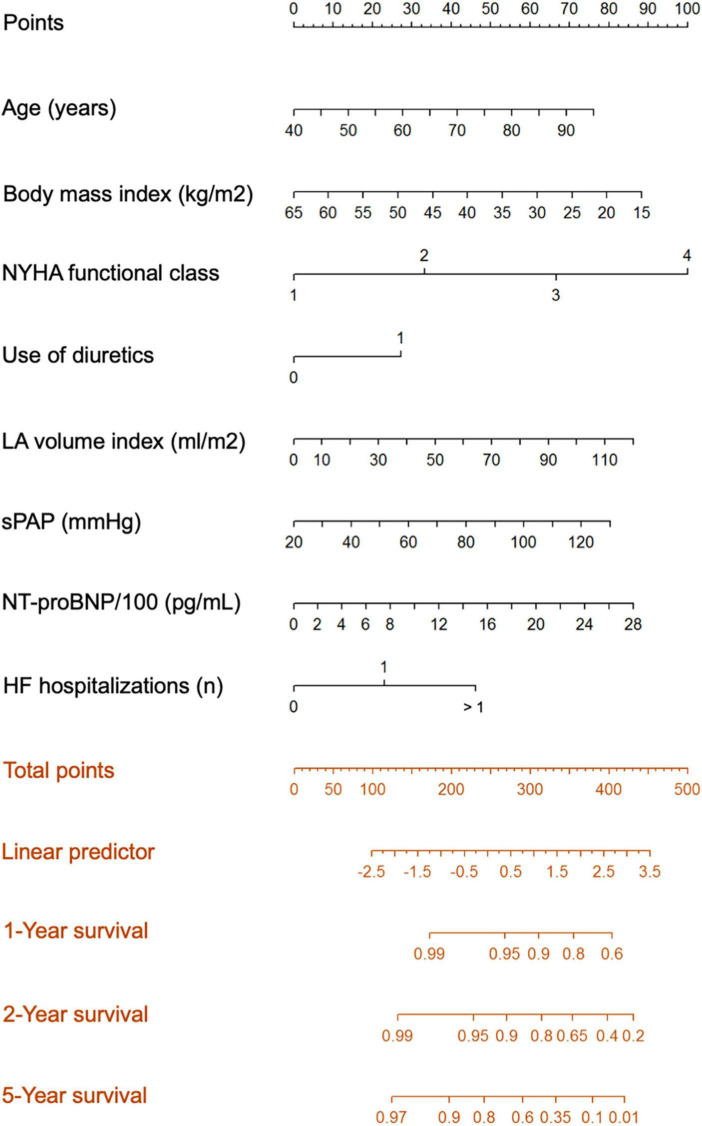
The Hfpef survivAL hOspitalization (HALO) prediction model for all-cause mortality.

To better understand the impact of acute HF events, we built a model to predict the outcome of patients with HFpEF. 16 clinical variables were tested as predictors of interest based on medical considerations. Additional variables of interest related to hospitalizations before baseline were the number of hospitalizations before baseline categorized as no, one, or more than one hospitalization and the time interval between the last hospitalization and baseline. HALO score obtained from augmented backward elimination showed a significant predictive capability for all-cause survival and retained the following variables: age (*p* = 0.081), BMI (*p* = 0.124), NYHA functional class (*p* = 0.004), need for diuretic therapy (*p* = 0.06), LAVI (*p* = 0.048), sPAP (*p* = 0.013), NT-proBNP (*p* = 0.076), and status of previous hospitalizations (*p* = 0.006; Table 2). The predictive model is illustrated by a nomogram showing the contribution of each variable to the score of the Cox regression model (Figure 3). A subanalysis of cardiac deaths (*n* = 64) yielded a comparable predictive model, retaining all variables except for BMI, which was superseded by COPD (Supplementary Table 3).

**TABLE 2:** Prediction model for all-cause death (*n* = 107).

HALO (Hfpef survivAL hOspitalization) prediction model			

Variables	Hazard ratio	Confidence interval	*P*-value
Age (years)	1.024	0.997–1.051	0.081
Body mass index (kg/m^2^)	0.971	0.934–1.008	0.124
NYHA functional class	1.761	1.197–2.588	0.004
Use of loop diuretic therapy	1.589	0.980–2.576	0.061
LA volume index (ml/m^2^) (IQR)	1.012	1.000–1.025	0.048
Pulmonary arterial systolic pressure (mmHg)	1.013	1.003–1.022	0.013
NT-proBNP/100 (pg/ml)	1.054	0.995–1.116	0.076
Category of HF^†^ hospitalizations	1.481	1.115–1.968	0.006

NYHA, New York Heart Association; LA, left atria; NT-proBNP, N-terminal prohormone of brain natriuretic peptide; HF, heart failure.

^†^HF hospitalizations were categorized in no, one and more than one episode defined by the concomitant presence of the symptom of sudden-onset dyspnea and clinical signs of acute cardiac decompensation, including weight gain and fluid retention, with presence of pulmonary or peripheral edema requiring intravenous diuresis.

Given a strong association between the number of hospitalizations after baseline and overall survival (log-rank test *p* < 0.001), the derived score was then used in an ordinal logistic regression model to predict future HF hospitalizations. The fitted model had an odds ratio of 3.24 (95% CI: 2.45–4.37, *p* < 0.001; Table 3), which means that with an increase of 1 in the HALO score, the odds of being at least once hospitalized against not being hospitalized at all increase by a factor 3.24 and the same holds true for the odds of having more than one hospitalization against having not more than one hospitalization. Supplementary Figure 2 illustrates the HALO score predictive probabilities for subsequent hospitalizations according to the distribution of the three different categories of hospitalizations.

**TABLE 3:** Ordinal logistic regression for HF hospitalizations and validation of the HALO score.

HALO (Hfpef survivAL hOspitalization) score*					


	Coefficient	Standard error	Hazard ratio	Confidence interval	*P*-value
Ordinal logistic regression for HF hospitalizations (*n* = 422)	1.18	0.15	3.24	2.45–4.37	**<0.001**
Validation cohort (*n* = 75)	0.76	0.25	2.13	1.30–3.47	**0.003**

*The HALO score obtained from augmented backward elimination has been derived to predict overall survival in patients with heart failure and preserved ejection fraction and was subjected to external validation in an independent cohort.

Bold indicates *p* < 0.05.

### Validation of the risk calculator

In a final step, we tested the performance of the score in an independent cohort of 75 HFpEF patients with comparable patient characteristics and prognostic factors (Supplementary Table 2). Using the score from the derivation data set as predictor of survival we obtained a coefficient of 0.76 indicating a discrimination that is slightly worse than in the original data set and a significant prediction of survival with a HR of 2.13 (95% CI: 1.30–3.47, *p* = 0.002; Table 3).

**FIGURE 4:**
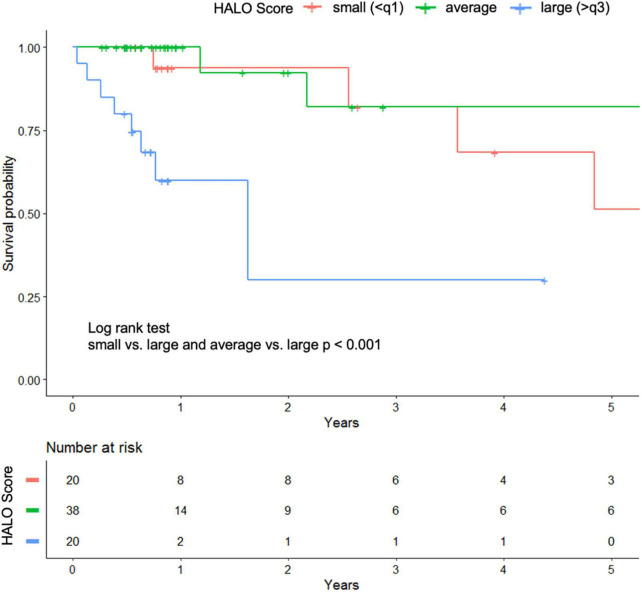
Kaplan–Meier plot of the validation cohort stratified by quartiles of the HALO score.

In the Kaplan–Meier analysis, overall survival was stratified into quartiles based on the HALO score derived from the validation cohort. This shows a visually perceptible discrimination of the model in predicting lower survival in quartile 3 with the highest risk (Figure 4).

### Clinical scenarios

Table 4 illustrates how to use the nomogram from Figure 3 to obtain 2-year survival probabilities for two fictitious clinical patients. A 75-year-old obese patient with NYHA IV dyspnea on loop diuretic therapy, left atrial enlargement, and elevation of systolic pulmonary pressure, a measured NT-proBNP level of 2,850 pg/ml, and five previous hospitalizations for HF would achieve 423 points in the nomogram, which then translates into a HALO score of approximately 3. This score results in a predicted 2-year survival of 20%. A patient of the same age and BMI who is asymptomatic with a normal LAVI and normal sPAP, an NT-proBNP level of 600 pg/ml, and one previous hospitalization for HF decompensation would achieve 217 points in the nomogram, which corresponds to a HALO score of approximately -0.5 and a predicted 2-year survival slightly higher than 95%.

**TABLE 4:** Clinical scenarios.

	Characteristics		Points
Case 1	Age (years)	75	48
	Body mass index (kg/m^2^)	34	55
	NYHA functional class	IV	100
	Use of diuretics	yes	29
	LA volume index (ml/m^2^)	40	30
	sPAP (mmHg)	54	25
	NT-proBNP/100 (pg/ml)	2850	88
	HF hospitalizations (*n*)	5	48
	Total points		423
	Linear predictor		3
	Survival		20% 2-Year survival
Case 2	Age (years)	75	48
	Body mass index (kg/m^2^)	34	55
	NYHA functional class	I	0
	Use of diuretics	yes	29
	‘LA volume index (ml/m^2^)	30	22
	sPAP (mmHg)	46	20
	NT-proBNP/100 (pg/ml)	600	18
	HF hospitalizations (*n*)	1	25
	Total points		217
	Linear predictor		1.2
	Survival		80% 2-Year survival

NYHA, New York Heart Association; LA, left atrial; sPAP, pulmonary arterial systolic pressure; NT-proBNP, N-terminal prohormone of brain natriuretic peptide; HF, heart failure.

## Discussion

In the present study, recurrent HF hospitalizations were associated with a significantly higher clinical risk profile, more signs and symptoms of congestive HF, and an increased risk of long-term mortality. In contrast to chronic HF, acute HF events lead to further deterioration of cardiac function, especially at very high end-diastolic pressures with further activation of neurohormones (19). Data from large trials and recent registries have shown that most hospitalizations for acute HF occur because of congestion (rales, jugular venous distension, edema) rather than a low cardiac output (20, 21). Although congestion is thought to begin as a compensatory mechanism in response to reduced cardiac output, clinical and experimental data suggest that congestion actually could contribute to the progression of HF (22). Likewise, in our study, acute HF events were found to worsen preexisting HF. There was a short-term improvement after each admission due to acute HF; however, the patient left the hospital with higher NT-proBNP serum levels as compared to pre-admission.

The diagnosis of HFpEF is difficult to affirm in the outpatient setting. Misclassification of non-specific symptoms such as dyspnea (23), challenges on physical examination due to obesity, venous stasis, and chronic lung disease (24), and variable, comorbidity-dependent disease markers (natriuretic peptide levels) (25) all contribute to diagnostic complexity. As HFpEF patients represent a very heterogeneous population, it is difficult to identify patients with an increased risk of death or cardiovascular events. Clinical factors such as impaired right ventricular function (26) and high left ventricular filling pressures (27) have previously been linked to outcome and recurrent hospitalizations. Yet, accurate quantification of right ventricular function is challenging, and direct measurement of filling pressures is limited due to its invasiveness. In contrast, our proposed prediction model is based on readily available parameters, which facilitate its practical application. After extensive adjustment by backward elimination, the combination of the variables age, BMI, NYHA functional class, need for diuretic therapy, LAVI, sPAP, NT-proBNP level, and status of previous hospitalizations was found to offer the best prediction for overall survival. In addition, use of the HALO score demonstrated strong predictive power for future acute HF events and, most importantly, the prognostic model proved satisfactory discrimination and prediction accuracy in the validation sample.

HFpEF is a heterogeneous clinical syndrome that is the result of risk factors that ultimately lead to abnormal cardiac structure and function, which in turn cause decreased cardiac output or increased cardiac filling pressure (28). Phenomapping of patients with HFpEF resulted in mutually exclusive groups of individuals with related comorbidities and pathophysiologies; the phenogroups identified had different outcomes, indicating different risk profiles and clinical courses (29). Based on this knowledge, we generously included comorbidities as potential risk-modifying factors in our analysis. Surprisingly, most of them did not indicate increased patient risk. However, consistent with the three phenotypes described by Shah et al., the inclusion of patient age, BMI, NT pro-BNP level, use of loop diuretics, left atrial volume, and presence of pulmonary hypertension provide optimal parameters for risk stratification in the heterogeneous collective of HFpEF patients. In addition to NYHA class, a history of previous HF hospitalizations most strongly influences the risk of patients with HFpEF and should therefore be included in individual risk stratification and further management.

Whereas most of the variables appear to be very conclusive and reflect the clinical profile of HFpEF patients very well, BMI showed an unexpected result. Patients with lower BMI seem to have an increased risk for mortality and future hospitalizations. Obesity represents one of the most common comorbidities in patients with HFpEF (30), and previous studies indicate the negative impact of morbid obesity on cardiac remodeling and the development of HFpEF (31). However, there is not yet agreement on the relationship between BMI and outcome in HFpEF. The I-PRESERVE trial and other large trials showed that mildly overweight patients had the lowest rates of death or HF hospitalizations (30, 32). Both underweight and severely overweight groups had higher all-cause mortality than slightly overweight patients. The underlying mechanisms are not fully understood, but adiponectin, an adipocyte-specific cytokine, seems to be involved in the observed effects (33). We can confirm the observation of the obesity paradox with our model, showing that patients with a lower BMI are at higher risk.

Besides NYHA functional class, repeated hospitalizations for HF were the strongest factor influencing outcome. It is anticipated that precisely these patients will benefit the most from the substance classes recently tested in large trials (4, 10). In the PARAGON-HF trial, treatment effects of sacubitril/valsartan compared with valsartan appeared to be amplified when initiated in patients who were recently hospitalized (34). The use of the SGLT-2 inhibitor empagliflozin in the EMPEROR-Preserved study revealed a significant reduction in hospitalization rates, which was consistently seen across all predefined subgroups, with a particularly pronounced effect seen in previously hospitalized patients (10).

Taken together, we have shown that HFpEF patients who experience recurrent HF hospitalizations have an unfavorable long-term outcome. Acute HF events contribute significantly to worsening of existing HF. Extensive efforts should therefore be made to keep HFpEF patients compensated over a longer period of time. Now that drugs are available for the first time that are likely to be of particular benefit to high-risk patients, it is even more important to identify patients at increased risk for mortality and recurrent events. The model presented here is simple and has been shown to be useful not only in risk stratifying survival, but can also be used to predict future HF hospitalizations.

### Study limitations

Although the current study provides valuable information on the impact of recurrent HF hospitalizations and ultimately led to the development of an easy-to-use risk stratification tool, it is not without limitations. First, due to the single-center nature, a center-specific bias cannot be excluded. However, there are some major advantages in limiting data collection to a single center: (a) inclusion of a homogenous patient population, (b) adherence to a constant clinical routine, (c) comprehensive clinical work-up with invasively measured confirmation of elevated LV filling pressures, (d) constant follow-up of the patient cohort. Second, in contrast to serially collected NT-proBNP values, other parameters such as echocardiographic follow-up or functional tests performed over time, such as 6MWTs or cardiopulmonary exercise testing, as well as medication adjustments made during the course, were not fully available and could have provided further information on the influence and progression of recurrent HF events. Furthermore, including other echocardiographic parameters on their risk prediction in the model would certainly have been interesting; however, because of the sum of adverse events, the number of variables that could be integrated was limited. Third, the validation cohort is rather small which limits the reliability of validation due to sampling variability. Nevertheless, the group of patients is very well characterized and comparable to the derivation cohort with results suggesting good discrimination of high-risk patients. Forth, whether these high-risk patients will benefit from intensified therapy and novel treatment approaches remains to be seen.

## Conclusion

We developed and validated a simple model to better identify HFpEF patients at increased risk of death and HF hospitalizations. Besides NYHA class, recurrent HF hospitalizations were the strongest driver of outcome. Intensive efforts should therefore be made to maintain HFpEF patients compensated over time.

## Data availability statement

The datasets presented in this article are not readily available because the data is the property of the Medical University of Vienna. For inquiries, please contact the Legal Department. Data can only be forwarded after positive approval by the legal department and the head of the department. Requests to access the datasets should be directed to datenclearing@meduniwien.ac.at.

## Ethics statement

The studies involving human participants were reviewed and approved by Ethics Committee of the Medical University of Vienna. The patients/participants provided their written informed consent to participate in this study.

## Author contributions

LS and DB: conceptualization, investigation, and funding acquisition. LS, FF, and DB: formal analysis, methodology, validation, visualization, and writing—original draft. BS, RR, T-MD, CB, FD, and DD: data curation. RB-E and JK: resources. BS, RR, T-MD, CB, FD, DD, RB-E, JK, and CH: writing—review and editing. All authors contributed to the article and approved the submitted version.
