# Supplementary material for: Heart failure with preserved ejection fraction: Calculating the risk of future heart failure events and death
Source: Front Cardiovasc Med. 2022 Oct 21;9:921132. doi: 10.3389/fcvm.2022.921132 (PMC9634582; doi:10.3389/fcvm.2022.921132)
Supplement: Supplementary file 1 [file Data_Sheet_1.docx]

Supplementary Material

# Supplementary Data

# Supplementary Figures

**Supplementary Figure 1.** **Flow chart**


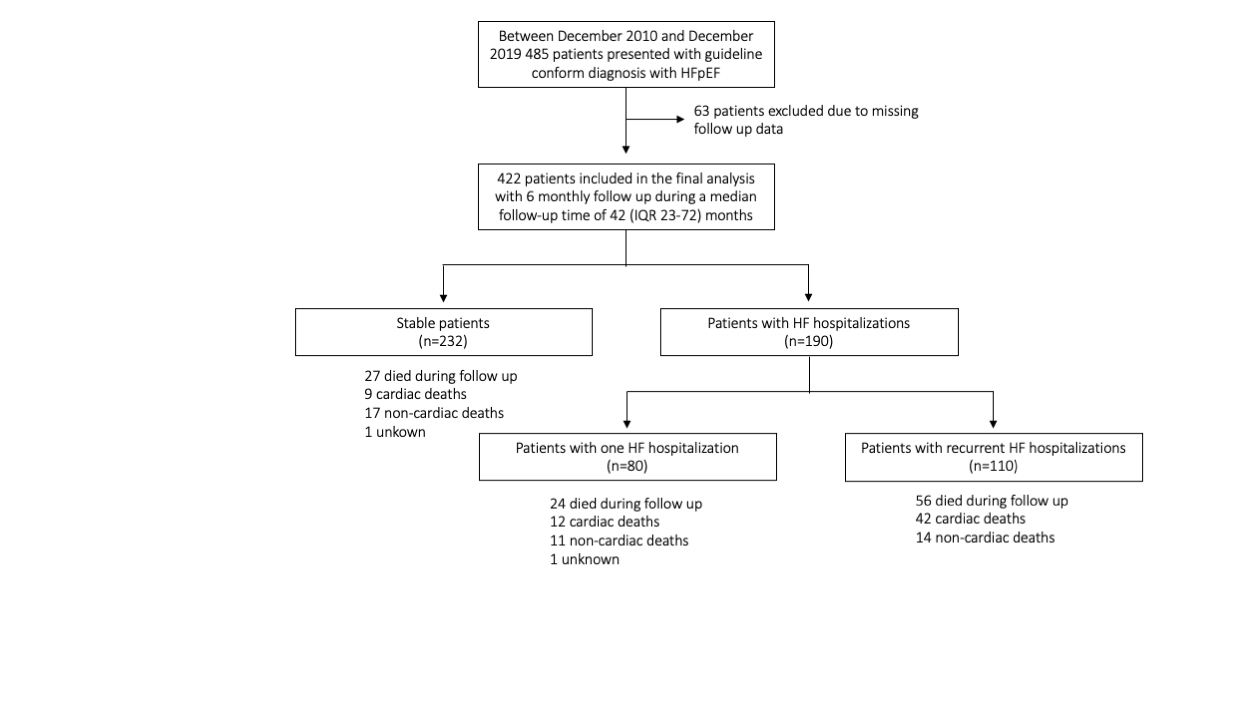


**Supplementary Figure 2.** **Prediction probabilities of future heart failure hospitalizations**

Score from the prediction model stratified by the number of heart failure hospitalizations.


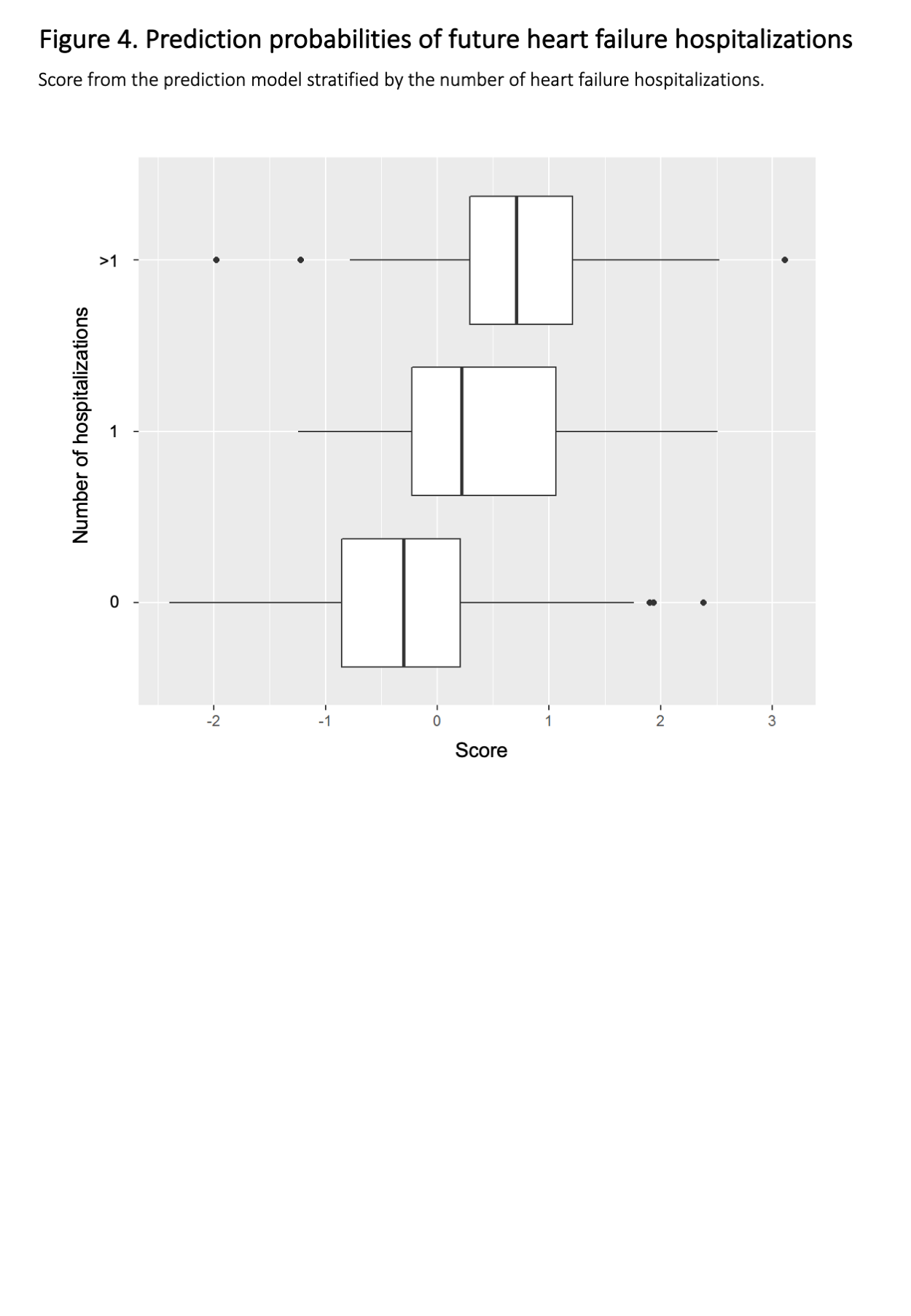


## Supplementary Tables

# Supplementary Table 1. Co-medication of registered patients with one or recurrent episodes of acute heart failure requiring hospitalization.

|  | **Stable patients**  **(n=232)** | **Patients with one HF hospitalization†**  **(n=80)** | **Patients with recurrent HF hospitalizations†**  **(n=110)** | ***p* value^‡^** | ***p* value**  **1 vs >1 HF hospitalizations^$^** |
| --- | --- | --- | --- | --- | --- |
| Calcium channel blockers, n (%) | 62 (26.8) | 31 (38.8) | 28 (25.5) | 0.086 | 0.051 |
| Beta blockers, n (%) | 168 (72.7) | 55 (68.8) | 86 (78.2) | 0.328 | 0.142 |
| Angiotensin-converting enzyme inhibitors, n (%) | 72 (31.2) | 24 (30.0) | 30 (27.3) | 0.764 | 0.681 |
| Angiotensin II receptor blockers, n (%) | 96 (41.6) | 32 (40.0) | 39 (35.5) | 0.559 | 0.523 |
| Diuretics, n (%) | 161 (69.7) | 68 (85.0) | 103 (93.6) | **<0.001** | 0.050 |
| Loop diuretics, n (%) | 90 (39.0) | 57 (71.3) | 90 (81.8) | **<0.001** | 0.086 |
| Thiazide diuretics, n (%) | 63 (27.3) | 25 (31.3) | 30 (27.3) | 0.776 | 0.551 |
| Mineralocorticoids, n (%) | 79 (34.2) | 32 (40.0) | 66 (60.0) | **<0.001** | **0.006** |

HF, Heart failure

Values are given as median and interquartile range (IQR), or total numbers (n) and percent (%). Bold indicates p<0.05.

† HF hospitalization was defined by the concomitant presence of the symptom of sudden-onset dyspnea and clinical signs of acute cardiac decompensation, including weight gain and fluid retention, with presence of pulmonary or peripheral edema requiring intravenous diuresis.

‡ For comparisons Chi-square test was used for categorical and Kruskal wallis 1-way ANOVA for continuous variables.

$ For comparisons Chi-square test was used for categorical and Mann-Whitney U-test for continuous variables.

# Supplementary Table 2. Baseline characteristics and prognostic factors in the derivation cohort and the validation cohort.

|  | **Derivation cohort**  **n=422** | **Validation cohort**  **n=75** | **P value** |
| --- | --- | --- | --- |
| **Clinical parameters** | | | |
| Age, years (IQR) | 74 (68-77) | 76 (69-80) | 0.084 |
| Female gender, n (%) | 274 (70.1) | 57 (73.1) | 0.595 |
| Body mass index, kg/m^2^ (IQR) | 29 (25-34) | 28 (26-33) | 0.415 |
| 6-minute walk distance, m (IQR) | 347 (253-423) | 329 (207-404) | 0.546 |
| NYHA functional class ≥ III, n (%) | 231 (60.5) | 12 (63.2) | 0.815 |
| NT-proBNP, pg/mL (IQR) | 1068 (416-1979) | 973 (573-2130) | 0.595 |
| HF hospitalization† prior to study inclusion, n (%) | 123 (31.5) | 31 (39.7) | 0.155 |
| Loop diuretic therapy, n (%) | 220 (56.3) | 49 (62.8) | 0.285 |
| **Co-morbidities** | | | |
| Arterial hypertension, n (%) | 369 (94.4) | 69 (88.5) | 0.055 |
| Atrial fibrillation, n (%) | 232 (59.3) | 51 (65.4) | 0.319 |
| Diabetes mellitus, n (%) | 135 (34.5) | 18 (23.1) | 0.049 |
| Chronic kidney disease*, n (%) | 200 (53.9) | 40 (51.9) | 0.754 |
| Anemia, n (%) | 173 (44.2) | 37 (50.0) | 0.362 |
| Chronic obstructive pulmonary disease, n (%) | 106 (27.2) | 18 (23.1) | 0.454 |
| **Echocardiographic parameters** | | | |
| LA indexed for BSA, ml/m2 (IQR) | 39 (29-51) | 36 (30-50) | 0.986 |
| LV-ejection fraction, % (IQR) | 59 (54-65) | 60 (54-64) | 0.619 |
| Pulmonary arterial systolic pressure, mmHg (IQR) | 49 (36-64) | 48 (32-67) | 0.846 |

NYHA, New York Heart Association; NT-proBNP, N-terminal prohormone of brain natriuretic peptide; HF, Heart failure; ALAT, Alanin Aminotransferase; ASAT, Aspartat Aminotransferase; Gamma-GT, Gamma-Glutamyl Transferase; GFR, Glomerular Filtration Rate; LDH, Lactatdehydrogenase; HbA1c, Glycated hemoglobin; LA, Left atrial; RA, Right atrial; LV, Left ventricular; E/E’, ratio of peak early transmitral flow velocity to peak early diastolic mitral annulus velocity; E/A, ratio of peak early transmitral flow velocity to mitral peak velocity of late filling; TAPSE, Tricuspid annular plane systolic excursion.

Values are given as median and interquartile range (IQR), or total numbers (n) and percent (%). Bold indicates p<0.05.

* Estimated glomerular filtration rate <60mL/min/1.73m^2^

† HF hospitalization was defined by the concomitant presence of the symptom of sudden-onset dyspnea and clinical signs of acute cardiac decompensation, including weight gain and fluid retention, with presence of pulmonary or peripheral edema requiring intravenous diuresis.

**Supplementary Table 3. Prediction model for cardiac death (n=63)**

| HALO (Hfpef survivAL hOspitalization) prediction model | | | |
| --- | --- | --- | --- |
| Variables | Hazard Ratio | Confidence Interval | *p*-value |
| Age | 1.035 | 0.998-1.074 | 0.065 |
| Chronic obstructive pulmonary disease | 1.711 | 0.955-3.066 | 0.071 |
| NYHA functional class | 2.559 | 1.574-4.160 | **<0.001** |
| Use of loop diuretic therapy | 1.687 | 0.879-3.236 | 0.116 |
| LA volume index, mL/m2 | 1.022 | 1.007-1.037 | **0.003** |
| Pulmonary arterial systolic pressure, mmHg | 1.018 | 1.005-1.031 | **0.007** |
| NT-proBNP/100, pg/mL | 1.403 | 0.987-1.995 | **0.004** |
| Category of HF^†^ hospitalizations | 1.535 | 0.651-1.057 | **0.024** |

NYHA, New York Heart Association; LA, Left atrial; NT-proBNP, N-terminal prohormone of brain natriuretic peptide; HF, Heart failure

† HF hospitalizations were categorized in no, one and more than one episode defined by the concomitant presence of the symptom of sudden-onset dyspnea and clinical signs of acute cardiac decompensation, including weight gain and fluid retention, with presence of pulmonary or peripheral edema requiring intravenous diuresis
